# Beyond autophagy: LC3-associated phagocytosis and endocytosis

**DOI:** 10.1126/sciadv.abn1702

**Published:** 2022-10-26

**Authors:** Carolina Peña-Martinez, Alexis D. Rickman, Bradlee L. Heckmann

**Affiliations:** ^1^ Department of Molecular Medicine, USF Morsani College of Medicine, Tampa, FL, USA.; ^2^ Byrd Alzheimer’s Center, USF Health Neuroscience Institute, Tampa, FL, USA.

## Abstract

Noncanonical functions of the autophagy machinery in pathways including LC3-associated phagocytosis and LC3-associated endocytosis have garnered increasing interest in both normal physiology and pathobiology. New discoveries over the past decade of noncanonical uses of the autophagy machinery in these distinct molecular mechanisms have led to robust investigation into the roles of single-membrane LC3 lipidation. Noncanonical autophagy pathways have now been implicated in the regulation of multiple processes ranging from debris clearance, cellular signaling, and immune regulation and inflammation. Accumulating evidence is demonstrating roles in a variety of disease states including host-pathogen responses, autoimmunity, cancer, and neurological and neurodegenerative pathologies. Here, we broadly summarize the differences in the mechanistic regulation between autophagy and LAP and LANDO and highlight some of the key roles of LAP and LANDO in innate immune function, inflammation, and disease pathology.

## Teaser

LAP and LANDO diverge from autophagy and regulate inflammation.

## INTRODUCTION

**Fig. 1.**
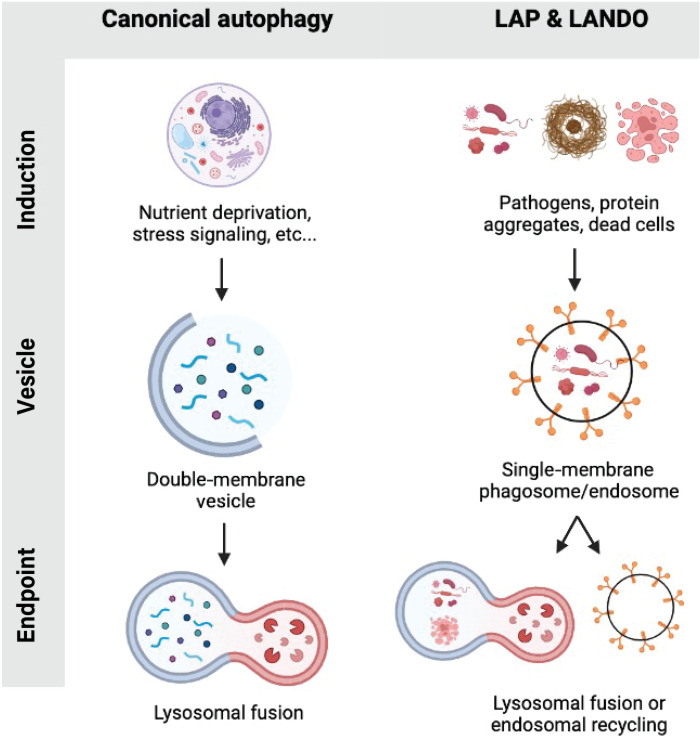
Comparison of canonical autophagy and LAP and LANDO. Graphical overview of select activation stimuli, vesicle dynamics, and pathway endpoint (lysosomal fusion or recycling) are illustrated.

Macroautophagy, autophagy or canonical autophagy henceforth, is an evolutionarily conserved process for either the nonspecific engulfment of cytoplasmic material or specific targeted engulfment of damaged organelles as well as misfolded or aggregated proteins into double-membrane autophagosomes (*1*). Subsequent trafficking of the autophagosome and fusion with lysosomes results in the degradation of the engulfed material (*2*–*4*). As such, autophagy functions as a mechanism of cellular homeostasis and recycling. Upstream signals including nutrient deprivation, intracellular stress signaling, and growth signaling result in the activation of a multistep cascade governed by the autophagy-related proteins (ATGs) and culminating in the lipidation of the microtubule-associated protein light chain 3 (LC3/ATG8) to phosphatidylethanolamine (PE) residues in the developing autophagosome or phagophore membrane (*2*–*7*). Recent studies have shown that the machinery that governs the processing and conjugation of LC3 in autophagy can be used in similar yet distinct molecular pathways, collectively referred to as noncanonical autophagy or noncanonical functions of the autophagy machinery (*8*–*14*). A subset of the autophagy machinery is required for LC3/ATG8 lipidation to single-membrane vesicles, a process known as conjugation of ATG8 to single membranes (CASM) (*15*) in pathways including LC3-associated phagocytosis (LAP) and LC3-associated endocytosis (LANDO), while other components typically required for autophagy are dispensable as will be reviewed herein (Fig. 1) (*16*–*21*).

In addition to differences in the autophagy machinery as will be discussed, the source of cargo is a major defining factor delineating autophagy from LAP and LANDO. As mentioned, autophagic cargo is derived from intracellular sources, whether that be intracellular debris, damaged organelles, or intracellular pathogens (Fig. 1). In stark contrast, cargoes that initiate LAP and LANDO are not intracellular in nature and stem from the extracellular environment (Fig. 1). Dead or dying cells are activating stimuli for LAP, while protein aggregates and receptor ligation events have been shown to promote LC3 lipidation in LANDO (*16*, *17*).

The physiological roles for the conjugation of LC3 to single membranes have best been characterized in the context of LAP. As will be expounded upon herein, LAP is activated in response to phagocytic cargoes including dead cells following recognition by efferocytic receptors such as T cell immunoglobulin and mucin domain containing 4 (TIM4) in macrophages (*22*). Further studies have found important roles for LAP in the recognition and clearance of multiple pathogens and further roles in regulating antigen presentation to adaptive immune cells. Abrogation of LAP results in heightened inflammatory signaling and inflammatory cytokine production upon stimulation. Like LAP, LC3 lipidation in LANDO promotes vesicle and receptor recycling events, and pathway abrogation leads to increased inflammation. LANDO was originally identified in microglia, innate immune cells of the central nervous system (CNS), in a model of Alzheimer’s disease (AD). LANDO protects mice against aberrant neuroinflammation and facilitates the recycling of receptors that are responsible for recognizing and clearing β-amyloid (Aβ) (*17*). Together, these functions of LANDO restrict pathology in murine models of AD.

In the present review, we will delineate the mechanistic differences between canonical autophagy and LAP and LANDO. Furthermore, we will summarize the functions of LAP and LANDO in immune regulation and inflammation in the context of a variety of settings including dead cell clearance, pathogen exposure, and neurodegenerative disease.

## LAP AND LANDO: A MECHANISTIC DIVERGENCE FROM CANONICAL AUTOPHAGY

### Shared and distinct proteins, membrane lipids, and timing

Canonical autophagy is activated by a diverse set of signals including nutrient deprivation or starvation, cellular stress, and other factors and is found in essentially every cell type (*23*). This self-cannibalism is a highly conserved process characterized by the sequestration of the cytoplasmatic cellular content into an endoplasmic reticulum (ER)–derived autophagophore with subsequent vesicle maturation and ultimately fusion with lysosome for cargo degradation. This process is regulated by the ATG proteins. ATG proteins regulate the formation of the autophagosome in a hierarchical manner through a cascade of phosphorylating and ubiquitin-like conjugating events (*14*).

Upstream signaling known to activate autophagy is governed by nutrient sensing primarily through the mammalian target of rapamycin (mTOR) complex 1 (mTORC1) and its inhibition, which results in the recruitment and activation of the autophagy machinery (*19*, *24*). Upon nutrient deprivation, adenosine monophosphate (AMP)–activated kinase (AMPK) is activated and simultaneously inhibits mTOR, leading to the assembly of the autophagic preinitiation complex. This complex is required for autophagy activation and is composed of proteins including Unc-51–like autophagy-activating kinase 1 (ULK1), FAK family kinase–interacting protein of 200 kDa (FIP200), and ATG13 (*25*, *26*). ULK1 phosphorylates and recruits the transmembrane protein ATG9 and class III phosphatidylinositol 3-kinase (PI3KC3) complex. The core components of PI3KC3 complex are Beclin1 and the PI3K VPS34, as well as associated factors including nuclear receptor–binding factor 2 (NRBF2) and activating molecule in beclin-1–regulated autophagy (AMBRA1) (*27*, *28*). ULK1 phosphorylates AMBRA1, initiating translocation to the ER and subsequent autophagosome formation. The autophagy PI3KC3 complex produces phosphatidylinositol 3-phosphate [PI(3)P], which acts as a recruitment signal for the downstream ubiquitin-like conjugation systems: the ATG5-ATG12 system and the ATG8-LC3-PE conjugation system (*29*). Both systems are required for the curvature, elongation, and sealing process of the autophagosome, as well as the lipidation of LC3 to PE in the autophagosome membrane (*29*, *30*).

Following LC3 decoration and autophagosomal sealing, LC3 interacts with microtubules via a variety of motor and motor adaptor proteins including PLEKHM1, PLEKHM2/SKIP, FYCO1, and JIP1 and kinesins and dyneins, leading to trafficking of the sealed autophagosome to lysosomes for fusion (*31*–*34*). In addition to motor proteins and motor adaptors, small guanosine triphosphatases (GTPases) including RAB7 and RAB2 as well as SNARE complexes including STX17-SNAP29-VAMP7/8 are key components in the tethering and fusion of LC3^+^ vesicles to lysosomes (*35*, *36*). Most of these events are likewise dependent on specific phosphoinositide species present in the LC3^+^ autophagosome including PI(3)P.

In contrast to autophagy, the conjugation of LC3/ATG8 to single-membrane vesicles in LAP and LANDO occurs downstream of receptor ligation events on the cell surface. Dissimilar to autophagy, the autophagy preinitiation complex containing FIP200/ULK1 is completely dispensable for both LAP and LANDO activation (*17*, *19*, *20*). Likewise, signaling through pathways including mTOR and AMPK does not lead to the activation of LAP or LANDO (*19*–*21*). The PI3KC3 complex that is found in the autophagy pathway is required for LAP and LANDO; however, the composition of the complex is unique in LAP and LANDO. The LAP/LANDO PI3KC3 complex is distinct from the complex used in canonical autophagy. Conserved proteins found in both complexes include Beclin1, VPS34, and VPS15 (*19*, *20*). As observed in canonical autophagy, VPS34 is the catalytic subunit in the complex that phosphorylates phosphotidylinositides to produce PI(3)P. Similarly, VPS15 is a pseudokinase that regulates VPS34 activity (*19*, *37*, *38*). As with the autophagy PI3KC3 complex, Beclin1 regulates the VPS34 lipid kinase function. However, unlike its sister complex in autophagy, the LAP/LANDO PI3KC3 complex requires the ultraviolet radiation resistance-associated gene protein (UVRAG) and Rubicon (*19*, *20*, *39*). These proteins take the place of AMBRA1 and ATG14L that are found in the autophagy PI3KC3 complex, both of which are dispensable for LAP/LANDO activation (*19*, *20*). Of particular importance is Rubicon, a RUN domain–containing protein that has been previously shown to inhibit PI(3)P production and LC3 lipidation events in canonical autophagy (*40*). Interestingly, deficiency of Rubicon reduces the generation of PI(3)P by VPS34 on phagosomal and endosomal membranes following LAP/LANDO activation, ultimately influencing downstream production of reactive oxygen species (ROS) and LC3 lipidation (*19*, *20*). ROS production is required for LAP and is reviewed further below.

Another contrasting aspect that mechanistically delineates LAP and LANDO from autophagy are the variances in timing with respect to the production of PI(3)P. During LAP and LANDO, VPS34 produces PI(3)P in the outer leaflet of the phagophore/endosome following cargo encapsulation and vesicle sealing (*19*, *20*). In autophagy, VPS34 production of PI(3)P occurs at specific foci on the ER in the earliest steps of autophagosome biogenesis, even proceeding cargo engulfment (*19*, *20*, *41*). In the context of LAP and LANDO, the nature of PI(3)P production following phagosome sealing suggests that the activity of VPS34 and the Rubicon-VPS34-Beclin1 complex in general is not required for cargo recognition and engulfment. Moreover, it further suggests that internalization of the phagosome is independent of PI(3)P production, consistent with other reports (*19*, *20*). In concordance with this idea, during the phagocytic clearance of apoptotic bodies in *Caenorhabditis elegans*, an event that engages LAP, the overexpression of a PI(3)P phosphatase that dephosphorylates PI(3)P to PIP is sufficient in abrogating the maturation of the phagosome without altering phagosome formation (*19*, *20*, *42*). During autophagy, the opposite is observed whereby VPS34 activity aids in localizing the autophagophore to the specific site where it will eventually elongate and engulf cytoplasmic cargo (*43*, *44*). Unlike what is observed during LAP and LANDO, dephosphorylation of PI(3)P following overexpression of PI(3)P phosphatase impairs the formation and assembly of the autophagosome (*45*).

Membrane PI(3)P can serve as a platform for the recruitment of a variety of effector proteins, many of which use PX and FYVE domains to interact with PI(3)P. The main PI(3)P effector proteins found in the autophagy pathway are the WIPI family of proteins, which alternatively bind PI(3)P using the WD β-propeller PROPIN lipid binding domain (*46*–*48*). Now, synonymous with autophagic function, WIPI2 simultaneously binds PI(3)P and ATG16L1. Binding of WIPI2 to ATG16L1 links two major signaling moieties in autophagy, the PI3KC3 complex and the lipidation machinery (*49*, *50*). However, while WIPI2 is required for autophagy, it is completely dispensable for LAP (*19*, *50*) and most likely for LANDO, although this remains to be directly tested. Distinct PI(3)P effector proteins in LAP and LANDO have not been identified; however, early endosome antigen 1 (EEA1) has been shown to participate in phagosome maturation and contains a PI(3)P binding FYVE domain (*51*). Whether EEA1 or similar proteins including those that participate in the autophagy pathway function in LAP and LANDO requires further investigation.

### ROS and NADPH oxidase

Another point of divergence between autophagy and LAP is the requirement for ROS production in LAP (*19*–*21*). In macrophages, nicotinamide adenine dinucleotide phosphate (NADPH) oxidase-2 (NOX2) generates ROS during phagosome maturation (*19*, *20*). NOX2 is a multiprotein complex with both membrane-associated and cytosolic subunits that produces a superoxide anion, which is rapidly converted to hydrogen peroxide in the phagosome lumen, and this and other forms of ROS can cause oxidative damage, particularly relevant to the killing of phagocytosed pathogens (*19*, *20*, *52*). Certain bacteria have developed methods for inactivating NOX2 by expressing proteins that disrupt NOX2 function, thereby circumventing phagosomal clearance. Genetic depletion of NOX2 in macrophages abrogates the lipidation of LC3 following LAP stimulation using either zymosan or apoptotic cells but has no effect on autophagy activation (*19*–*21*). NOX2 is the predominant isoform of the NOX proteins found in most monocytic cells including macrophages. Interestingly, other innate immune cells including microglia have robust expression of other NOX isoforms such as NOX4 (*53*). To date, no studies have evaluated the role of these other NOX isoforms in LAP regulation; however, on the basis of their ability to produce ROS, they likely function similarly to NOX2. Furthermore, the requirement for ROS and NOX2 function is likely conserved between LAP and LANDO; however, it has not been investigated. ROS production is observed during receptor recycling and other endocytic processes; therefore, it is reasonable to hypothesize that ROS is required for LANDO; however, further evaluation is necessary.

### The role of LC3 in LAP and LANDO

**Fig. 2.**
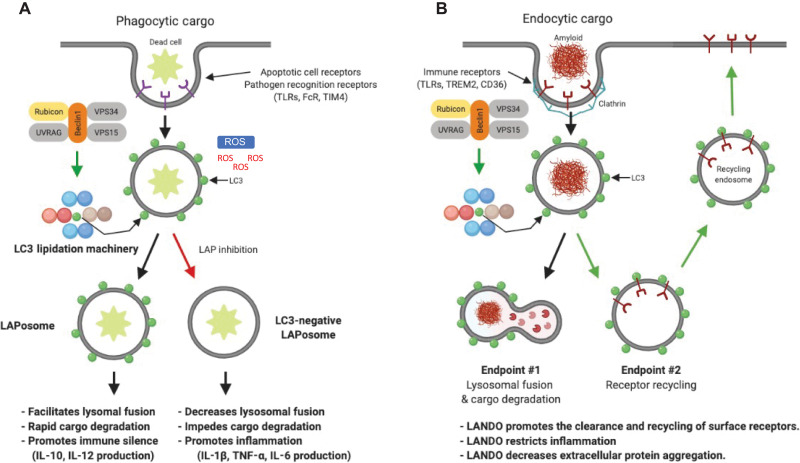
Comparison of LAP versus LANDO. (**A**) Phagocytic cargo such as apoptotic cells activates LAP. The LAP/LANDO PI3KC3 complex including Rubicon is assembled, leading to LC3 lipidation onto the sealed phagosome. The LC3^+^ LAPosome facilitates cargo degradation and restricts inflammatory immune responses. Abrogation of LC3 lipidation leads to defective cargo clearance and increases inflammation. (**B**) Endocytic cargo including protein aggregates binds to cognate receptors including TLRs and TREM2, leading to clathrin recruitment and endosome internalization. The LAP/LANDO PI3KC3 complex containing Rubicon is assembled, and LC3 lipidation occurs in a similar manner to LAP. Unlike LAP, LANDO activation leads to multiple endpoints including cargo degradation in the lysosome in addition to the recycling of receptors to the plasma membrane. LANDO promotes the recycling of a subset of cell surface receptors including TLR4, TREM2, and CD36 and restricts inflammatory immune activation. LANDO impairment leads to extracellular accumulation of cargo due to decreased surface receptors and promotes robust inflammatory cytokine production.

While it is evident that there are shared components between LAP, LANDO, and autophagy, there is an appreciable amount of divergence and unique regulators found in LAP and LANDO (Fig. 2). Differences in the production of PI(3)P, the components of the PI3KC3 complex, and the need for ROS production, as highlighted above, further delineate these CASM pathways from canonical autophagy. Interestingly, the downstream functions of LC3 lipidation to the phagosome in LAP appear to be quite consistent with the functions observed in canonical autophagy. LC3 lipidation aids in autophagosome trafficking and directs autophagosome/lysosome fusion events as well as other functions including cargo selection in canonical autophagy. In LAP, LC3 lipidation occurs on the outer face of the phagosome membrane following full phagosome closure. LC3 conjugation in autophagy occurs on both faces of the double-membrane autophagophore before elongation and closure (*54*). Interestingly, by the time the autophagosome has matured to closure, most conjugated LC3 resides on the outer surface of the autophagosome, similar to what is observed on the phagosome in LAP (*19*, *20*). Expression of the *Legionella* effector protein RavZ, which specifically cleaves lipidated LC3 from both double and single membranes, was sufficient in delaying the expression of the lysosomal marker LAMP1 following LAP activation (*19*, *20*). These data hint at a putative role for lipidated LC3 in phagosome-lysosome fusion, consistent with canonical autophagy. In contrast to both autophagy and LAP, the role of LC3 lipidation in the context of LANDO is poorly defined. We can hypothesize that LANDOsomes destined for degradation follow similar principals as those observed for both autophagosomes and LAPosomes. In the view of receptor recycling and endosomal sorting back to the plasma membrane, LC3 lipidation is likewise obligatory. Overexpression of RavZ or a dominant-negative mutant of ATG4, both leading to either delipidation or inhibition lipidation of LC3, respectively, decreases the recycling of receptors including triggering receptor expressed on myeloid cells 2 (TREM2), Toll-like receptor 4 (TLR4), and CD36 (*17*). These data show that in either scenario, LC3 lipidation is required. The mechanisms by which cells sort endosomes that are LC3^+^ to either the lysosome, plasma membrane, or other cellular locales remain elusive. It is plausible that autophagy adaptor proteins such as p62 are interacting with the LANDOsome and other endocytic regulators including Rab7 to facilitate trafficking (*55*). The RabGAP protein TBC1D5, which is centric to endosomal trafficking, interacts with both LC3 and ATG9 (*56*, *57*). Furthermore, both TBC1D5 and ATG9 have been shown to interact with the AP2 complex and clathrin, and disruption of TBC1D5 or clathrin-mediated endocytosis leads to accumulation of ATG9 in late endosomes (*56*, *57*). It is not unimaginable that other unique adaptors exist, which aid in “directing” LC3^+^ endosome trafficking in the context of both LANDO and autophagosome/endosome fusion events.

### New insights: Posttranslational modification of Rubicon

Recent evidence has suggested that posttranslational modifications (PTMs) of Rubicon alter its role in regulating LAP activation and may possibly aid in delineating LAP from LANDO. The intracellular bacteria *Listeria monocytogenes* is able to suppress LAP by modulating mitochondrial Ca^2+^ signaling, leading to production of acetyl–coenzyme A (acetyl-CoA) (*58*). Rubicon was found to be acetylated in response to increased acetyl-CoA levels, decreasing LAPosome formation (*58*). Global acetylation was unaffected, suggesting that acetyl-CoA is accumulating in a mitochondria-phagosome compartment and is responsible for the preferential modification of Rubicon (*58*). These findings have further implications especially given the compartmentalization model proposed, as it suggests that compartment-specific PTMs could direct or modulate Rubicon function to specific membrane compartments, such as phagosomal versus endosomal membranes. Moreover, diverse acetylation events are known mediators of receptor recycling and endosomal sorting, leading credence to further implications of PTMs in altering Rubicon function across multiple pathways (*59*).

**Fig. 3.**
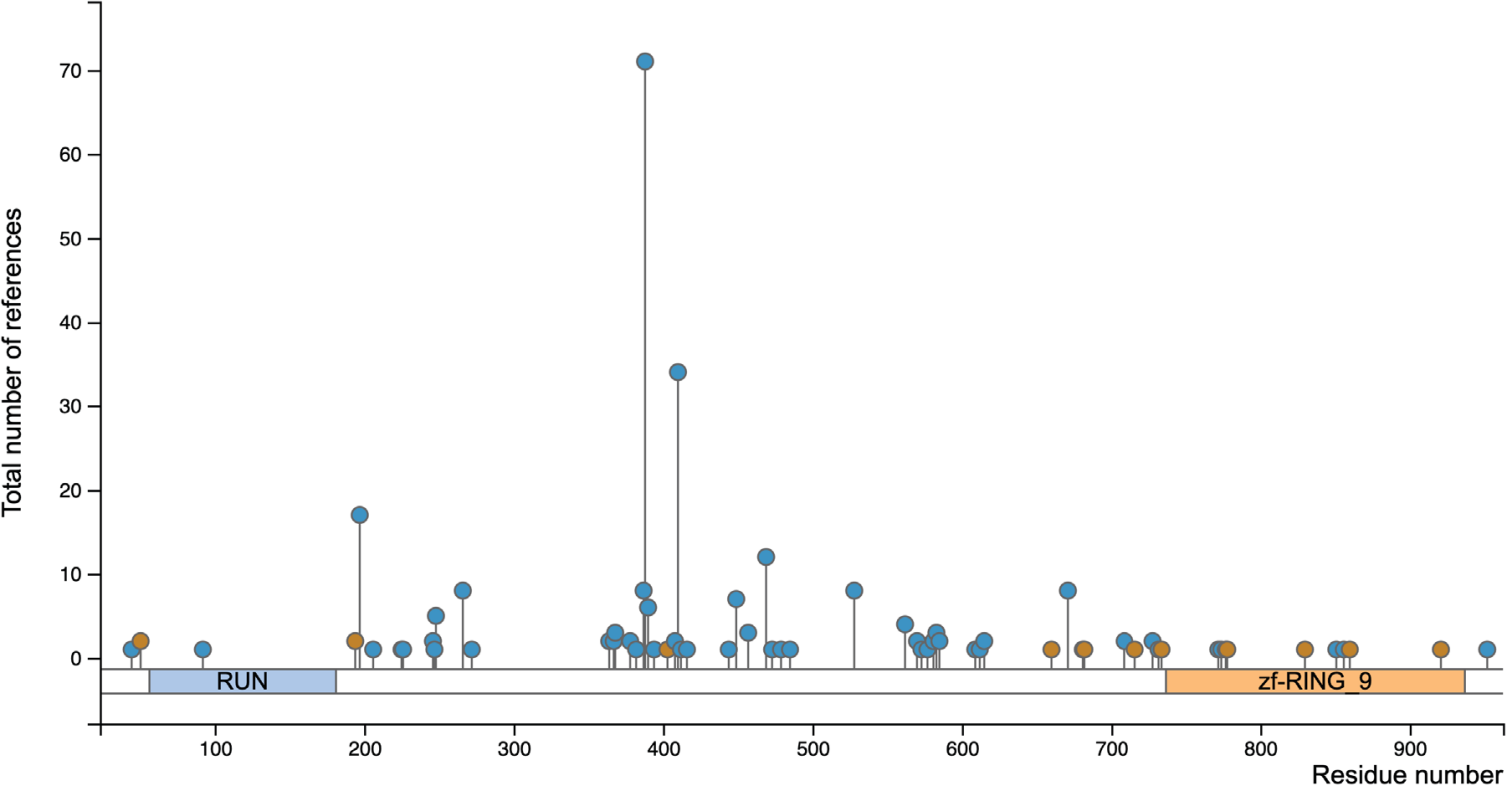
PhosphoSite analysis of human Rubicon indication over 50 putative phosphorylation sites. The number of literature references corresponding to each putative site is plotted on the *y* axis.

In addition to acetylation, Rubicon has multiple putative phosphorylation sites including serine-44 and serine-92 in the N-terminal region, which are phosphorylated by hormonally up-regulated Neu-associated kinase (HUNK) (*60*). Phosphorylation of Rubicon by HUNK promotes VPS34 activity and canonical autophagy (*60*). The activation of LAP and LANDO was not explored, but it would be interesting to determine whether there is a preference of the dephosphorylated form of Rubicon of sequestration toward the LAP/LANDO PI3KC3 complex over the autophagy complex. Phosphorylation-deficient mutants of Rubicon that prevent HUNK-mediated phosphorylation suppressed autophagic VPS34 activity and PI(3)P production (*60*). We speculate further that this and possibly other phosphorylation events could be defining factors in controlling Rubicon between autophagy and LAP/LANDO. PhosphoSite analysis of human Rubicon alone reveals over 50 putative phosphorylation sites including serine-44/92 (Fig. 3). Given the ability for phosphorylation to modulate not only protein-protein interactions but also protein-membrane interactions (*61*, *62*), it is plausible that a role for phosphorylation in the association of Rubicon with specific lipid species in distinct membrane structures including phagosomes and endosomes exists. Future studies directed at further defining these and other PTMs are of importance for improving our understanding of the intricate cell biology associated with Rubicon.

## MASTERING IMMUNITY: THE ROLE OF LAP AND LANDO IN INNATE IMMUNE ACTIVATION AND INFLAMMATION

To date, the best characterized functions of LAP and LANDO in governing innate immune function are in the context of inflammatory signaling and, to a lesser extent, in the processing and presentation of pathogenic antigen. LAP and LANDO modulate anti- and proinflammatory cytokine production in response to immunological stimuli including dead cells, a process termed efferocytosis, extracellular pathogens including bacteria and fungi, as well as aberrant extracellular protein aggregates including the AD peptide Aβ. As expected, this diverse set of stimuli known to engage either LAP or LANDO stems both from physiological scenarios, such as the clearance of dead cells following routine turnover, and under pathological settings, including neurodegenerative diseases and cancer.

### Taking out the trash: LAP in efferocytosis, pathogen recognition, and inflammatory implications

**Fig. 4.**
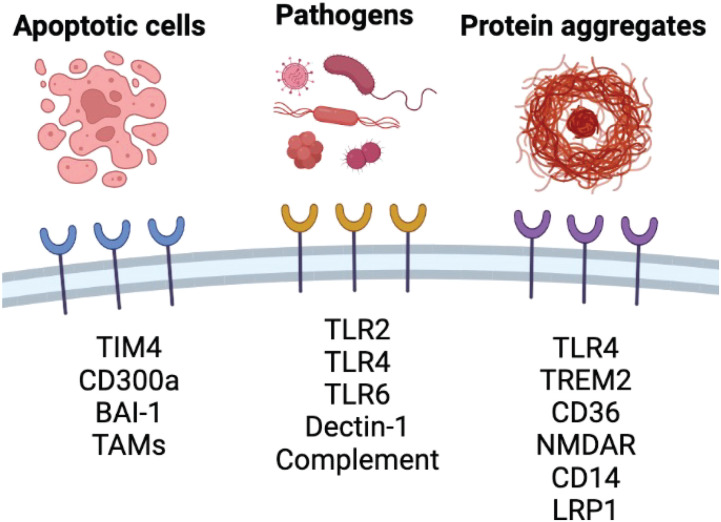
Overview of select receptors involved in the recognition of phagocytic and endocytic cargoes including apoptotic/dying cells, extracellular pathogens, and protein aggregates, which subsequently activate LC3 lipidation in LAP and LANDO.

The phagocytic clearance of apoptotic corpses, a process termed efferocytosis, is of paramount importance to maintaining tissue homeostasis (*21*, *63*). LAP is centric to this process and functions to limit innate immune cell activation following the recognition and engulfment of dead cell cargo (*19*, *20*). Upon death, dying cells produce a variety of “eat me” signals that are subsequently sensed by innate immune cells including macrophages. A primary eat me signal is the rearrangement of the membrane lipid phosphatidylserine (PS) from the inner to outer leaflet of the plasma membrane, a process characterized originally during apoptosis that is governed by the cleavage of flippases including ATP11C and scramblases such as Xkr8 by activated executioner caspases including caspase-3 (*64*). These events lead to functional switching of both protein classes leading to the flipping of PS to the outer leaflet and extracellular exposure (*64*). Once cells have translocated PS, it can be recognized by a group of receptors expressed on macrophages including CD300a, BAI-1, and TIM4 (Fig. 4). Binding of PS to TIM4 is a well-known activator of both efferocytic clearance and LAP. Upon engulfment of the dead cell following TIM4 binding, the LAP effector Rubicon is recruited to the developing phagophore as detailed above (*22*, *65*, *66*). These events trigger the stepwise assembly of the LAP machinery culminating in LC3 conjugation to the phagosome. The mechanisms that lead to the recruitment of Rubicon to the phagosome remain elusive, but changes in membrane dynamics and composition are one plausible hypothesis. Accumulation of dead cells in LAP-deficient Rubicon knockout mice leads to increased systemic inflammation characterized by up-regulation of proinflammatory cytokines and chemokines including tumor necrosis factor–α (TNF-α), interleukin-1β (IL-1β), IL-6, CCL-2, CCL-3, and CXCL-10, as well as commensurate reductions in anti-inflammatory mediators such as IL-10 (*19*–*21*). This accumulation of dead cells in aged LAP-deficient mice leads to the development of a lupus-like disease and autoimmune syndrome (*19*–*21*). Interestingly, deficiency in canonical autophagy but sufficiency in LAP failed to recapitulate the establishment of autoimmunity, demonstrating a full divergence of LAP from autophagy in a pathological setting (*19*–*21*).

Like the contribution of LAP in suppressing autoimmunity, LAP functions within tumor-associated macrophages (TAMs) to facilitate dead cell clearance and mitigate inflammation and is, however, beneficial for the tumor microenvironment. LAP deficiency in TAMs promotes M1-like proinflammatory polarization and compromises tumor growth (*67*). Mice deficient in LAP regulators including Rubicon, Beclin1, ATG16L1, ATG5, and ATG7 have restricted tumor growth when injected with B16F10 cells (*67*). In contrast, deficiency in canonical autophagy through genetic deletion of FIP200, ATG14, or ULK1 failed to have any effect on tumor growth. Moreover, LAP deficiency induced a unique differential gene expression profile in TAMs including up-regulation of type I interferon (IFN) signaling and cellular response pathways to type I IFN and type I IFN production (*67*). Increases were also observed in the interferon regulatory factor (IRF) complex, stimulator of interferon gene (STING) complex, and nuclear factor κB (NF-κB) pathway (*67*). Up-regulation of STING and type I IFN in LAP-deficient TAMs resulted in a robust antitumor response (*67*). This response was found to be T cell dependent and suggests that LAP in TAMs suppresses antitumoral T cell function mainly by restricting STING-dependent type I IFN production.

Similarly, to dead cell clearance, pathogens ranging from bacteria such as *Legionella dumoffii*, *Burkholderia pseudomallei*, and *L. monocytogenes* to fungi including *Candida albicans* and *Aspergillus fumigatus* activate LAP upon recognition by innate immune cells. Surface receptor engagement by pattern recognition receptors (PRRs) such as TLRs is known to activate LAP (*68*–*70*). TLR1/2, TLR2/6, and TLR4 have now been shown to be key LAP receptors, leading to downstream events following extracellular sensing of diverse pathogen-associated molecular patterns (PAMPs) and opsonized foreign particles (*15*, *19*, *20*, *71*–*74*). Interestingly, not all TLRs function in the same fashion with respect to LAP activation. Ligation of TLR9 does not lead to LAP engagement (*19*, *20*, *75*). In this case, LAP was not required for the delivery of immunoglobulin G (IgG)–DNA complexes to TLR9 in plasmacytoid dendritic cells (*75*).

Most pathogenic microorganisms are found within LAP-targeted, single-membrane phagosomes following recognition by PRRs leading to infection of host cells. In the context of *L. monocytogenes* infection, bacterial recognition is initiated following complement opsonization and engagement of complement receptors 1 and 3 (*76*–*78*). A diverse set of pathogen surface receptors (Fig. 4), including TLR2 and CD14 on macrophages, has also been implicated in facilitating the phagocytic internalization of multiple pathogens including *L. monocytogenes* and activation of PI3K signaling, required for LAP activation (*79*, *80*). Furthermore, LAP induced by *L. monocytogenes* is dependent on NOX2 and ROS, consistent with mechanistic regulation of LAP in response to dying cells (*81*). Knockout of NOX2 or treatment of infected cells with the antioxidant diphenyleneiodonium (DPI) diminishes LC3 lipidation on *L. monocytogenes* containing phagosomes (*81*). It is important to note that the mechanistic recognition and LAP activation are not ubiquitously shared and are unique to individual bacterial species, although significant overlap in mechanisms is communal and reviewed well in detail previously (*20*, *67*).

Fungi including *C. albicans* and *A. fumigatus* are recognized by the phagocytic receptor Dectin-1 (*20*, *74*, *82*). Internalization of fungi via Dectin-1 leads to intracellular signaling mediated by the Dectin-1–associated Splee tyrosine kinase (Syk). This signaling modality results in increased ROS production simultaneous to Rubicon recruitment and LC3 processing (*20*, *74*, *82*). Consistent with responses to dead cells or bacterial stimuli, canonical autophagy regulators including FIP200 and ULK1 are completely dispensable for this process (*19*, *20*). LC3 lipidation on fungi containing phagosomes is a critical step in promoting the degradation of the pathogen cargo by the lysosome. Linkage between LAP and inflammatory mechanisms following fungal infection has been proposed. Up-regulation of IFN-γ–induced death-associated protein 1 (DAPK1) during *A. fumigatus* infection leads to up-regulation of LAP and inhibits the activation of NACHT, LRR, and PYD domain–containing protein 3 (NLRP3) (*83*). Furthermore, DAPK1 interacts with LAP regulators including Rubicon, Beclin1, and Atg7. A dependency for fungal cell wall composition has also been shown to determine whether LAP is activated in response to infection. Swelling of the fungal cell wall leads to the presentation of immunostimulatory β-glucans in fungi including *A. fumigatus*, leading to LC3 lipidation (*82*, *84*). In contrast, fungi with cell walls deficient in β-glucans but abundant in melanin do not engage the LAP machinery and are able to evade LAP-mediated degradation, limiting ROS production and phagosome maturation (*82*).

Parasites including *Toxoplasma gondii* and *Plasmodium vivax* also lead to LAP activation. Invading parasites are found in single-membrane vesicles that are LC3^+^ (*19*, *20*, *74*, *76*). Like canonical LAP described above, LC3 recruitment to phagosomes containing parasites was dependent on PI3K activity, Beclin1, and ATG5, whereas ULK1 was dispensable (*19*, *20*, *74*, *76*). These findings are consistent with LAP activation, although the roles of Rubicon and ATG16L were not evaluated; therefore, canonical LAP activation during parasite infection remains to be fully elucidated, although these data suggest that LAP is instrumental in the clearance of at least a subset of parasites.

In contrast to LAP-mediated clearance of extracellular pathogens, canonical autophagy activation can eliminate microorganisms through a group of autophagic adaptors, known as sequestosome 1–like receptors (SLRs), which are responsible for elimination of microorganisms from the cytosol (*85*). Upon pathogen invasion, PAMPs activate PRRs including the TLR superfamily as well as nucleotide-binding and oligomerization domain (NOD)–like receptors (NLRs) (*86*). These events occur early during infection, which, in turn, stimulates the assembly of the autophagy machinery within the cell. Similarly, autophagic activation also reduces or restricts pathogen-induced cellular damage. An example of this occurs upon infection with the intestinal virus such as murine norovirus (MNV). In the absence of ATG16L1, MNV triggers intestinal pathologies in mice through up-regulation of IFN-γ and TNF such as Crohn’s disease (*87*), and these findings can be extrapolated to human biology as well. Therapeutically increasing autophagy improves survival in a mouse model of sepsis by reducing the level of inflammation in the lungs (*88*, *89*). Therefore, autophagy not only is applicable to directed clearance of intracellular pathogens but also has a key role in reducing the adverse effects associated with infection (*21*). No roles for LAP in the clearance of intracellular pathogens have been described because of the source of cargo in LAP compared to autophagy, extra- versus intracellular.

Another important function of the autophagy machinery beyond roles in CASM in immune regulation to keep in mind is the regulation of cellular energetics through autophagic activation. Maintaining cellular metabolism and substrate availability is a key component to ensuring appropriate functioning of both innate and adaptive immune cells alike. Nutrient sensing by AMPK and mTOR is critical for maintaining a functional immune cell homeostasis and ultimately allowing for activation and differentiation (*90*). An example of this is monocyte-to-macrophage differentiation. When a monocyte has received a differentiation signal such as exposure to granulocyte-macrophage colony-stimulating factor (GM-CSF), autophagy activation is increased (*91*). Activation of the c-Jun N-terminal kinase (JNK) pathway promotes the release of Beclin1 from BCL-2 and allows for participation of Beclin1 in the autophagy pathway. Moreover, GM-CSF exposure reduces the cleavage of ATG5 in differentiating monocytes (*91*). The cleavage product of ATG5 is a known apoptotic stimulus that leads to monocyte death. Inhibition of autophagy in human primary monocytes using the PI3K inhibitor 3-methyladenine (3-MA) results in a reduction in monocyte differentiation upon stimulation with GM-CSF and decreases monocyte survival due to increased apoptotic death (*91*). Furthermore, macrophage autophagy regulates a variety of cross-talk mechanisms between innate and adaptive immune cells including cytokine production and antigen presentation. Many long-held facets of autophagy in innate-adaptive immune cell cross-talk may, in fact, be alternative LC3 lipidation pathways such as LAP; however, further investigation is necessary to clarify any delineation that may exist.

### Presenting the invader: LAP in antigen processing

Early studies investigating the role of ATG5 in immune regulation revealed a reduction in the presentation of extracellular antigen to CD4 T cells upon ATG5 ablation (*92*, *93*). Recent studies evaluating the function of ATG16L1 in autophagy identified a mutant of ATG16L1 lacking the WD domain that resulted in impaired LC3 lipidation in CASM pathways including LAP and LANDO but failed to alter LC3 lipidation in canonical autophagy (*39*). The ATG16L1 WD domain–deficient mouse model has proven useful in further delineating CASM pathways and their regulation from canonical autophagy and the impact of altering these pathways on organismal and cellular physiology (*94*, *95*). For example, the WD domain of ATG16L1 is necessary for the presentation of the murine major histocompatibility complex (MHC) class II H2-E alpha chain on the mouse MHC class II molecule H2-Ab (*94*). NOX2 was also shown to be required for efficient MHC class II presentation, further indicating a role for LAP in antigen presentation (*94*, *96*). In addition, ROS is likewise a key component in this process, as the ROS-insensitive ATG4B mutant that inhibits LAP decreased MHC class II presentation of *Candida* antigens following phagocytosis (*96*, *97*). While the distinct mechanisms linking LAP to MHC class II presentation remain obscure, it is plausible that changes in vesicle trafficking and lysosomal degradation when decorated with LC3 lead to altered degradation kinetics, allowing for increased immunogenicity of internalized antigens as suggested previously (*19*, *20*, *92*). Conversely, in the context of dead cells, LAP facilitates the rapid degradation of internalized dead cell cargo as described above, and this may hint at a divergent regulation of LAP in a cargo-dependent manner. Interestingly, few roles for LAP in MHC class I presentation have been described; however, inhibition of the LC3 conjugation machinery in dendritic cells and pancreatic tumor cells promoted the expression of MHC class I as well as costimulatory molecules (*98*–*100*). It is tempting to speculate that inhibition of LAP in this setting leads to increased immune activation; however, further interrogation is necessary, specifically in evaluating LAP regulators including Rubicon- and WD domain–deficient ATG16L1, as most studies to date have evaluated molecules that are shared between LAP and canonical autophagy including ATG5, ATG7, ATG16L1 (full length), and others (*19*, *20*). LAP-like processes including LANDO have been shown to regulate inflammatory responses (Fig. 2), particularly in the context of neurodegeneration, and will be expanded upon below.

### Recycling to survive: The role of LANDO in neuroinflammation

The discovery and characterization of the immune implications of LAP paved the way for uses of the autophagy machinery in distinct molecular pathways. LANDO was identified and found to be similar to LAP in regulation, yet distinct in cellular function (Fig. 2). Cross-talk between autophagic pathways and late endosomal pathways has long been characterized; however, direct lipidation of LC3 on developing endosomes was recently described in LANDO, another distinct CASM pathway. Lipidation of LC3 to endosomes following receptor-mediated endocytosis promotes the recycling of a subset of internalized receptors and was found to be a critical mechanism in maintaining immune cell function and mitigating pathology in a model of AD (*8*, *16*–*18*). In response to Aβ, brain immune cells internalize Aβ through both phagocytic and endocytic processes. Activation of LANDO was observed in response to Aβ in microglia, and vesicles containing Aβ were identified as clathrin-positive endosomes. Abrogation of LANDO resulted in an impairment in the recycling of Aβ receptors including TLR4, TREM2, and CD36 (Fig. 2). This distinct recycling compartment is vastly different than delivery of phagocytic material to the lysosome via the LAP pathway and represents a novel cellular mechanism.

Age-related diseases, including AD, have been associated with reduced levels of autophagy and decreases in the machinery that regulate both canonical autophagy and CASM pathways such as LAP and LANDO (*8*, *16*–*18*). The autophagy machinery in neurons and microglia plays a vital role in the clearance of aggregation-prone forms of misfolded proteins characteristic of major neurodegenerative diseases including AD. The role of the autophagy machinery in the brain during pathology not only is limited to age-related diseases but also has been shown to have roles in traumatic brain injury (TBI) and during ischemic stroke (*101*–*104*). While canonical autophagy regulates cellular function often from a metabolic and nutrient angle, CASM pathways, in particular LANDO, are directed at the recognition and clearance of immune perturbations including aggregated proteins (Fig. 2). Together, these mechanisms govern homeostasis and immune regulation in the brain. In the context of AD, a devastating neurodegenerative condition resulting in memory loss, cognitive dysfunction, and ultimately death, LANDO appears to be an important mechanism for restricting inflammation and facilitating the clearance of Aβ as mentioned above. Clinically, AD can be distinguished from other forms of dementia and cognitive impairment through histopathological markers including the deposition and formation of extracellular Aβ plaques and intracellular tau protein neurofibrillary tangles (NFTs). These hallmarks are accompanied by synaptic dysfunction, neuroinflammation, and mitochondrial dysfunction leading to neuronal loss. Although the exact pathogenesis of AD is not fully understood, it is believed that Aβ and the hyperphosphorylation of Tau, forming NFTs, are centric events in the establishment and progression AD pathology (*105*). Autophagy has been shown to participate in Aβ generation from the aberrant cleavage amyloid precursor protein (APP) and secretion of neurotoxic Aβ peptides as well as throughout multiple mechanisms in AD pathobiology (*106*–*114*). In the context of autophagy in regulating Aβ production, the deficiency in the autophagy machinery leads to extracellular accumulation of neurotoxic Aβ and neurodegeneration that can be exacerbated by deletion of the autophagy protein ATG7 in neurons or by abrogation of Beclin1 function (*115*, *116*).

Besides neuronal autophagy, inflammation is now known to be a critical factor driving the progression of AD. LC3 lipidation in LANDO in response to Aβ restricts plaque formation, mitigates microgliosis and neuroinflammation, and prevents neurodegeneration (*8*, *16*–*18*). Either global or specific inhibition of LANDO in microglia, the primary innate immune cell of the CNS, was found to grossly exacerbate disease pathology (*16*, *17*). LANDO deficiency in microglia led to robust increases in microglial activation and the production of proinflammatory cytokines including TNF-α, IL-1β, and IL-6 (*16*, *17*). Further studies found that LANDO deficiency alone was sufficient to drive the establishment of endogenous age-associated spontaneous AD-like pathology in mice with significant exacerbation of CNS immune activation and inflammation (*18*). In stark contrast, microglial-specific deletion of canonical autophagy through knockout of FIP200 failed to provide similar results, demonstrating that the observed findings were LANDO specific and not due to canonical autophagy activation (*16*, *17*). LANDO also supports receptor recycling and ensures the maintenance of a proper pool of plasma membrane receptors, at least in the context of TLR4, TREM2, and CD36 (Fig. 2). In addition, mounting evidence is suggesting that selective autophagy is likely contributing to mitigation of AD pathology. Recent studies have indicated that mitophagy inhibition contributes to neurodegeneration observed in a variety of murine models of AD (*117*, *118*). This has not only been demonstrated in animal models, but also mitophagic impairment has been observed in the hippocampus of AD patients (*119*, *120*). It is plausible that an impairment in the clearance of damaged mitochondria from neurons and glia not only leads to dysregulated signaling but also may result in the accumulation of ROS and other damage-associated molecular patterns (DAMPs), which may function to propagate microglial activation and, ultimately, neuroinflammatory signaling.

In addition to LANDO, there is likely a role for LAP in AD, although to the best of our knowledge this has not been investigated directly. It is highly likely that the efferocytic role of LAP is functioning in microglial and astrocytes to clear dead or dying neurons in the AD brain and suppress neuroinflammatory activation and microglial M1-like polarization. As was observed in systemic lupus-like disease in aged Rubicon-deficient mice (*19*, *20*), accumulation of dead neurons and cellular debris would exacerbate AD pathology, consistent with an impairment in LAP. Like canonical autophagy and LANDO, regulators of LAP are also down-regulated with age and further suppressed in AD patients, establishing a possible correlation to these unique pathways and AD pathology in humans (*18*, *114*, *121*–*123*). Further investigation in the context of human AD biology is needed to definitively link the observed functions of autophagy, LAP, and LANDO in the murine AD brain to human neurobiology.

Unlike LAP, LANDO is likely to contribute to the recycling of receptors in most cell types well beyond professional phagocytes and microglia of the CNS. Although no published work regarding the role of LANDO in the recycling of receptors in nonphagocytic immune cells exists, multiple groups are actively exploring these facets of LANDO biology. It is tempting to speculate on the relevance of LANDO well beyond that of LAP in governing not only immune function but also cellular homeostasis and signaling as a whole. Multiple studies to date have demonstrated a significant cross-talk between the endocytic pathway and the autophagy pathway (*124*, *125*), and it is plausible that many of these events are indeed mediated by LANDO and not canonical autophagy. Therefore, new avenues of investigation including whole “receptome” profiling for the dependence of specific receptor recycling on LANDO are needed.

## BRIDGING THE GAP: FROM LAP AND LANDO TO INFLAMMATION, A MECHANISTIC VOID

From dead cell and pathogen clearance to the recognition of aberrant protein aggregates and receptor recycling, LAP and LANDO play key roles in mitigating inflammation. As described above, deficiency in either pathway leads to the robust activation of inflammatory processes, including the predominant activation of NF-κB and NLRP3 inflammasome signaling and the subsequent production of TNF-α and IL-1β. This intertwined theme of cytokine production when CASM is inhibited in LAP and LANDO further strengthens the implication of the noncanonical autophagy machinery in immune regulation. However, a major void exists with respect to the mechanistic regulation of inflammatory signaling downstream of LAP or LANDO deficiency. Why does deficiency in either of these processes result in inflammatory activation?

### More data yield more questions: The autophagy machinery in inflammation

To begin to decipher this question, we recall that the autophagy machinery can control the production of cytokines through multiple mechanisms. Autophagy activation can suppress inflammatory responses following infection by preventing proinflammatory cytokine production. This anti-inflammatory function of autophagy was first observed in mice deficient in ATG16L1 in a model of Crohn’s disease, which resulted in an exacerbated production of IL-1β and IL-18 (*126*). Similar changes in cytokines were also observed in response to endotoxin upon ATG16L1 deficiency (*127*). These cytokines are typically produced via signaling through PRRs, which eventually leads to the formation of the inflammasome. The NLRP3 inflammasome is a molecular complex consisting of proteins including NLRP3, ASC, and NEK7 that becomes activated upon signs of cellular danger leading to caspase-1 activation and downstream cleavage of pro–IL-1β and pro–IL-18, which, upon conversion to their active forms, are release via cell death pathways including pyroptosis (*128*). Autophagy, or more specifically mitophagy, could prevent inflammasome activation by removing damage mitochondria leading to reduced release of mitochondrial-derived DAMPs and reduced production of ROS. The accumulation of ROS is a critical determinant leading to the activation of NLRP3 signaling and inflammasome assembly and activation (*128*). Similar to ATG16L1 deficiency, depletion of Beclin1 or LC3 results in increased activation of caspase-1 and secretion of IL-1β and IL-18 (*129*, *130*). In addition to possibly regulating inflammasome activity by mitophagy as highlighted above, autophagy has been shown to directly degrade inflammasome components and caspase-1 levels are reduced following autophagic activation (*129*, *130*). It is further tempting to speculate on the consequences of LAP or LANDO inhibition on ROS production. Because of the need for ROS produced by NOX2, at least in the LAP pathway, inhibition of LAP may result in a gross accumulation of ROS that could subsequently activate the NLRP3 inflammasome.

While most roles for the autophagy machinery in cytokine regulation appear to be critical for suppressing inflammatory cytokine production by regulating NLRP3 inflammasome activation, other studies have found that the autophagy machinery can contribute to the “unconventional” secretion of IL-1β, IL-18, and the alarmin HMGB1 under stress conditions, seemingly promoting inflammation (*131*). This unconventional secretion depends on ATG5 and on a specialized unconventional secretory regulator, GRASP (GRASP55), which not only is important in unconventional secretion but also affects the canonical starvation-induced autophagy in mammalian cells (*132*, *133*). The exact mechanisms surrounding the unconventional release of cytokines and the interplay of the autophagy machinery in this setting are poorly understood and require further investigation. It is possible that energetic or stress status of the cell could play a role in “switching” autophagy from an anti-inflammatory mediator to a proinflammatory entity through unconventional cytokine release. Similarly, unconventional regulation of pathways that govern the release of cytokines including pyroptotic activation as well as inflammatory cell death through necroptotic activation may contribute to cytokine escape during LAP and LANDO. Recent evidence has linked Rubicon to the regulation of necroptotic cell death activation. Rubicon deficiency was found to sensitize mice toward necroptosis in a model of renal ischemia reperfusion (*134*). This propensity to undergo inflammatory cell death may indeed be a contributing factor to the inflammation observed downstream of LAP and LANDO inhibition. Whether the observed effects of Rubicon deficiency on necroptosis are due to roles of LAP and LANDO remains to be evaluated. Previous work has, however, illustrated a link between necroptotic activation via TNF-related apoptosis inducing ligand (TRAIL) ligation and the autophagy machinery, which is mediated by p62 and recruitment of the necroptotic inducing protein RIPK1 (*135*). This may potentially indicate a role for p62 beyond autophagy and in the realm of LAP and LANDO as suggested above.

Furthermore, Rubicon has been shown to be a physiological feedback inhibitor of PRR signaling, preventing uncontrolled proinflammatory responses. Upon Dectin-1 or RIG-I–mediated activation, Rubicon exchanges binding partners from 14-3-3β to CARD9 in a stimulation-specific and phosphorylation-dependent manner, terminating PRR-induced cytokine production (*56*). However, this function of Rubicon is likely independent of its role in LAP and possibly LANDO, as the unique binding of Rubicon and CARD9 occurs following β-1,3-glucan stimulation or Sendai virus infection, and rapamycin or zymosan stimulation does not modified their interactions (*56*). This suggests that Rubicon dynamically changes binding partners from 14-3-3β to CARD9 in a specific stimulation–dependent manner and likely represents a unique mechanism of inflammatory control.

### Rubicon directing inflammasome assembly: A speculation

Although no data currently exist to support the direct interaction of Rubicon with any of the components of the NLRP3 inflammasome or the trafficking/assembly of the complex, Rubicon has been shown to control the localization of proteins not only in the endocytic pathway but also to various other organelles including mitochondria (*136*). Could Rubicon be key in altering the interaction of inflammasome components? This might not be too large of a stretch as p62 negatively regulates inflammasome function (*137*). While the role of p62 in this context is largely due to its role in targeting the NLRP3 components for autophagic degradation, it suggests that the possibility of other ATGs including Rubicon could be involved. The key NLRP3 adapter protein ASC contains a CARD domain similar to the one found in CARD9 (*138*). One intriguing hypothesis is that Rubicon controls ASC localization or interaction with NLRP3, by direct interaction, similar to the interaction observed between Rubicon and CARD9.

One may also consider that decreased cargo degradation in LAP or reduced recycling of receptors in LANDO may facilitate constitutive immune signaling from either the LAPosome or LANDOsome. If an inability to efficiently decrease immune signaling exists, acute inflammation can persist and might explain increases downstream of LAP and LANDO deficiency. For example, upon uptake of dead cells, LAP facilitates the efficient degradation of the internalized corpses. This degradation is stalled when there is a deficiency in not only Rubicon but also the LAP pathway as a whole. Compensatory inflammatory signaling may be activated to “deal” with this failure. Moreover, the loss of pathways including LAP not only results in proinflammatory cytokine production but also decreases in anti-inflammatory cytokine expression and production including IL-10 and transforming growth factor–β (TGF-β) (*19*–*21*). This discord between anti- and proinflammatory signaling can perpetuate continued signaling toward dysregulated inflammation. While no data clearly define these hypotheses, it is clear that these phenomena are not restricted to Rubicon deficiency alone; instead, they are due to loss of LAP or LANDO as inflammatory profiles are consistent with not only Rubicon deficiency but also loss of other key pathway regulators downstream including ATG16L, ATG5, and ATG7 (*19*–*21*).

## SUMMARY

Over the past 30 years since the characterization of the autophagy machinery by Ohsumi and colleagues (*139*), the roles for the autophagy machinery not only in the physiological functions of canonical autophagy in metabolism and cellular homeostasis but also in alternative functions of the autophagy machinery in unique cellular pathways including CASM are ever expanding. Moreover, new evidence is illustrating roles for CASM pathways including LAP and LANDO across multiple biological mechanisms ranging from debris clearance to modulation of cellular signaling and governance of immune functions. Distinct roles for these pathways are now being established in multiple tissues in the context of not only normal physiology but also relevance to pathological mechanisms including pathogen invasion, autoimmunity, neuropathology and neurodegeneration, and cancer. Moreover, new investigation into the role of aging in regulating both canonical autophagy and LAP/LANDO is revealing unique trends. The expression of components of the LAP and LANDO machinery including Rubicon has been shown to decrease with age and is further suppressed in the AD brain (*18*). This suppression in LAP/LANDO leads to robust neurological pathology in AD models reviewed herein. A similar phenomenon is observed in response to *Streptococcus pneumoniae* infection that engages LAP (*140*). The activation and LAP response diminish with age in the *S. pneumoniae* model and may contribute to age-related susceptibility (*140*). Together, these two studies highlight an important aspect of LAP/LANDO biology that until recently has been overlooked and that is the impact of age, not only on the function of each pathway but also on the contribution to the global innate immune response.

Looking forward, the establishment of new experimental techniques including single-cell sequencing and spatial transcriptomics will be invaluable for decrypting the intricate control and cross-talk between canonical autophagy and noncanonical functions of the autophagy machinery in pathways such as LAP and LANDO, the impact of aging on the regulation of these pathways, and their important and often obligatory roles in organismal biology.
